# Supplementary material for: igv.js: an embeddable JavaScript implementation of the Integrative Genomics Viewer (IGV)
Source: Bioinformatics. 2022 Dec 23;39(1):btac830. doi: 10.1093/bioinformatics/btac830 (PMC9825295; doi:10.1093/bioinformatics/btac830)
Supplement: btac830_Supplementary_Data [file btac830_supplementary_data.pdf]

igv.js: an embeddable JavaScript implementation of the Integrative Genomics Viewer (IGV)

**Supplementary Materials**

# 1. igv.js Quick Start

In this tutorial we will embed an igv.js instance displaying a reference sequence, ideogram, genome annotations, and tracks in a web page.

## Step 1: Create an html page

To insert an IGV instance in a page an empty container is required into which an igv “browser” instance will be inserted. A “div” element is commonly used. To begin, create an HTML file with a plain text or HTML editor of your choice and paste in the following snippet

```
<html>
<head>
    <title>igv.js embedding example</title>
</head>

<body>

<div id="igvDiv"></div>

</body>
</html>
```

## Step 2. Create an igv.js browser instance

The igv “browser” is an object in which your page can interact to define a reference genome, load tracks, navigate around the genome, and listen for user events. The browser object is initialized with the container div, in which it places itself, and a configuration object. In this step we will create an empty browser with no configuration.

```
<html>
<head>
    <title>igv.js embedding example</title>
</head>

<body>

<div id="igvDiv"></div>

<script type="module">

    import igv from "https://cdn.jsdelivr.net/npm/igv@2.13.6/dist/igv.esm.min.js"

    const config = {}

    igv.createBrowser(document.getElementById("igvDiv"), config)

</script>

</body>
</html>
```

At this point, opening the HTML file in a browser will result in a page like this

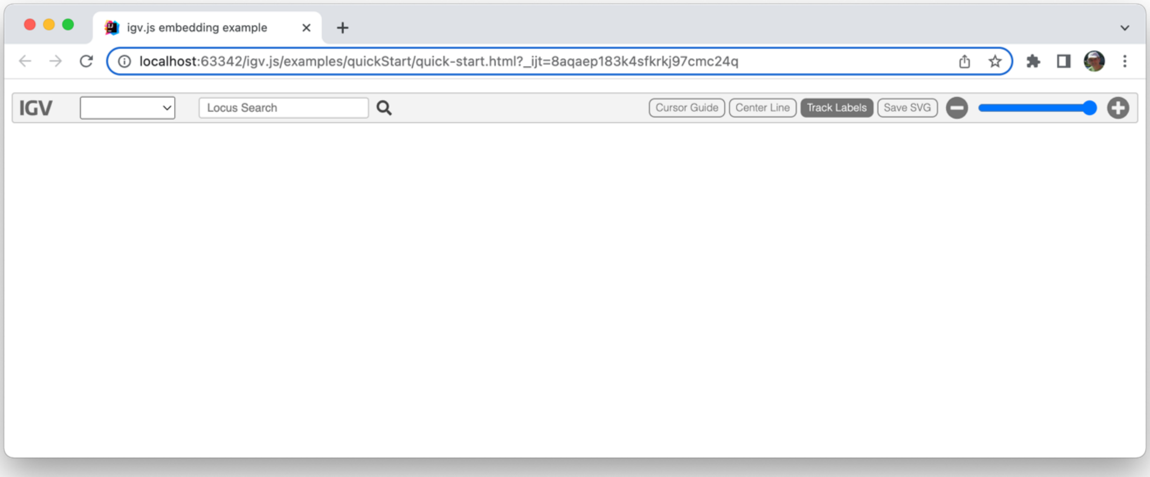


## Step 3. Specify a reference genome

Reference genomes are defined by URLs to a FASTA file and its index, and optionally URLs to a cytoband file for an ideogram, and a list of genome annotation tracks. This object is defined in detail in the documentation https://github.com/igvteam/igv.js/wiki/Reference-Genome. We will be using the Human assembly "hg38"

```
const config = {
    reference: {
        id: "hg38",
        fastaURL: "https://s3.amazonaws.com/igv.broadinstitute.org/genomes/seq/hg38/hg38.fa",
        indexURL: "https://s3.amazonaws.com/igv.broadinstitute.org/genomes/seq/hg38/hg38.fa.fai",
        cytobandURL: "https://s3.amazonaws.com/igv.org.genomes/hg38/annotations/cytoBandIdeo.txt.gz"
        tracks: [
            {
                "name": "Refseq Genes",
                "format": "refgene",
                "url": "https://s3.amazonaws.com/igv.org.genomes/hg38/ncbiRefSeq.txt.gz"
            }
        ]
    }
}
```

Alternatively, for genomes hosted by IGV, we can specify the reference by its identifier using the "genome" property. As hg38 is a hosted genome we will use this short form

```
    const config = {
        genome: "hg38"
    }
```

The complete list of IGV hosted genomes can be found here:
https://s3.amazonaws.com/igv.org.genomes/genomes.json

## Step 4. Specify an initial locus

Optionally we can also specify an initial locus for the browser view. We are interested in the MYC gene. This is specified with the “locus” property:

```
const config = {
    genome: "hg38",
    locus: "myc”
}
```

At this point our page will look like this this, with ideogram, sequence, and annotations.

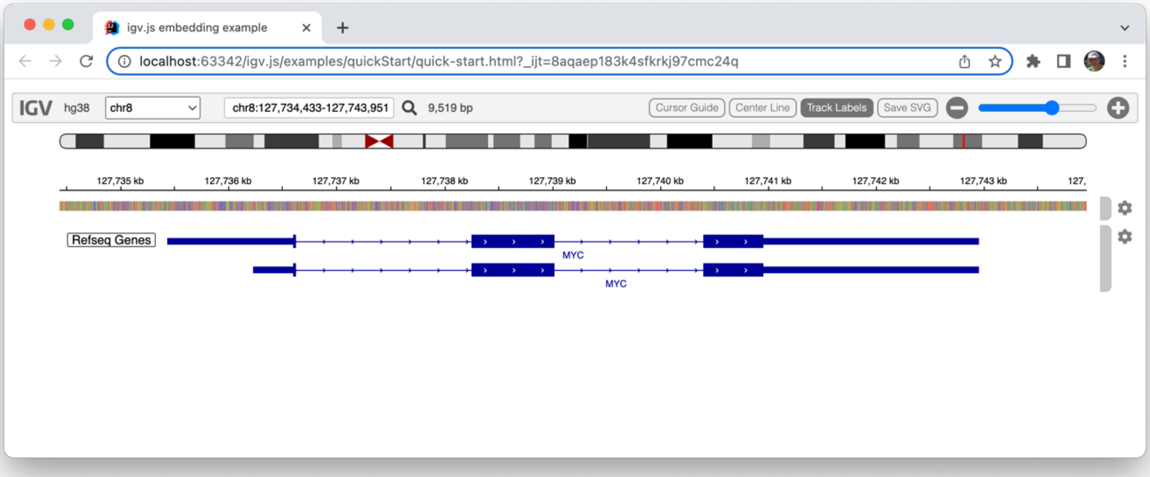


## Step 5. Specify an initial track collection

Tracks are specified as a list of configuration objects, each specifying a URL to the track data, a track type, and a file format. Many other properties can optionally be set and are described in the documentation at https://github.com/igvteam/igv.js/wiki. A summary of track types and associated file formats are listed below. In our example we will load two ChIP-Seq signal tracks from the ENCODE project.

```
tracks: [
        {
          name: "GM12878 H3K27ac ",
          url: "https://www.encodeproject.org/files/ENCFF716VWO/@@download/ENCFF716VWO.bigWig",
          color: "rgb(200,0,0)",
          type: "wig",
          format: "bigwig"
         },
         {
           name: "GM12878 H3K4me3 ",
           url: "https://www.encodeproject.org/files/ENCFF669DTI/@@download/ENCFF669DTI.bigWig",
           color: "rgb(0,150,0)",
           format: "bigwig",
           type: "wig"
        }
```

```
        ]
```

Our HTML page with an embedded igv.js browser is now complete

```
<html>
<head>
    <title>igv.js embedding example</title>
</head>

<body>

<div id="igvDiv"></div>

<script type="module">

    import igv from "https://cdn.jsdelivr.net/npm/igv@2.13.6/dist/igv.esm.min.js"

    const config = {
        reference: "hg38",
        locus: "myc",
        tracks: [
          {
            name: "GM12878 H3K27ac ",
            url: "https://www.encodeproject.org/files/ENCFF716VWO/@@download/ENCFF716VWO.bigWig",
                color: "rgb(200,0,0)",
                type: "wig",
                format: "bigwig"
          },
          {
            name: "GM12878 H3K4me3 ",
            url: "https://www.encodeproject.org/files/ENCFF669DTI/@@download/ENCFF669DTI.bigWig",
            color: "rgb(0,150,0)",
            format: "bigwig",
            type: "wig"
          }
        ]
    }

    const browser = await igv.createBrowser(document.getElementById("igvDiv"), config)

</script>

</body>
</html>
```

Opening this page in a browser

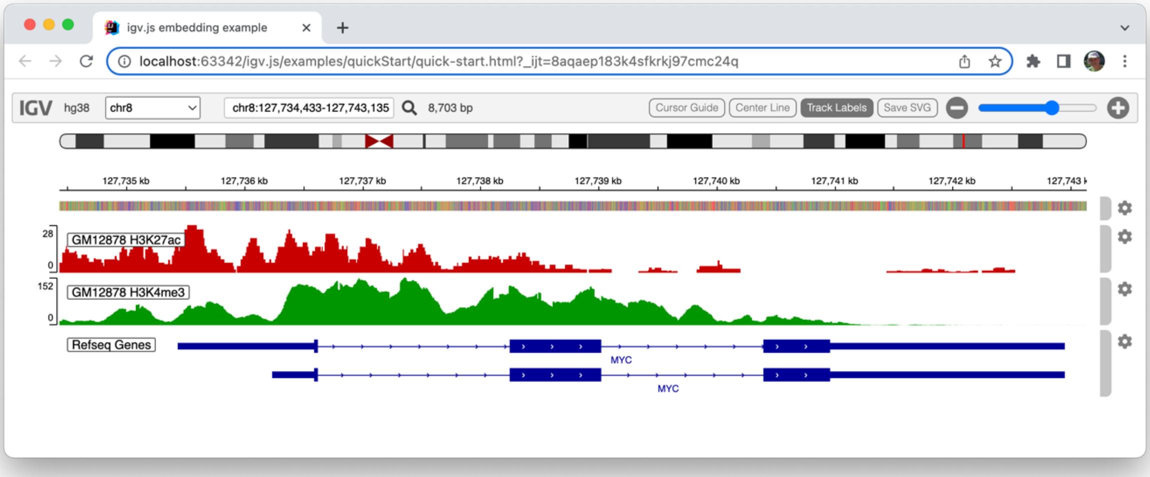

## 2. Track Types and File Formats

The table below lists supported track types and file formats for igv.js version 2.13.6.

| Track Type | Description | Formats* |
|---|---|---|
| sequence | Reference sequence | FASTA |
| alignment | Aligned sequence reads | BAM, CRAM |
| annotation | General annotations of the genome reference. Useful for representing a wide variety of entities including genes, transcripts, *-seq peaks, and general regions. | GTF, GFF, BED, bigBed, bigGenePred |
| wig | Aligned numeric scores, such as alignment coverage and *-seq signal values | WIG, BEDGRAPH, bigWig |
| seg | Segmented copy number values. | SEG |
| variant | Single nucleotide and structural variants. | VCF |
| mutation | Single nucleotide mutations | MAF |
| interact | Interactions between distal regions of the genome represented as arcs. | BEDPE, Interact, BigInteract |
| gwas | Association probabilities | GWAS |

*Format references:

FASTA: https://blast.ncbi.nlm.nih.gov/Blast.cgi
BAM: https://samtools.github.io/hts-specs/SAMv1.pdf
CRAM: https://samtools.github.io/hts-specs/CRAMv3.pdf
VCF: https://samtools.github.io/hts-specs/VCFv4.3.pdf
BED: https://samtools.github.io/hts-specs/BEDv1.pdf
GTF: https://uswest.ensembl.org/info/website/upload/gff.html
GFF: https://uswest.ensembl.org/info/website/upload/gff3.html
bigBed: https://genome.ucsc.edu/goldenPath/help/bigBed.html
bigGenePred: https://genome.ucsc.edu/goldenPath/help/bigGenePred.html
WIG: https://genome.ucsc.edu/goldenPath/help/wiggle.html
bigWig: https://genome.ucsc.edu/goldenPath/help/bigWig.html
BEDGRAPH: https://genome.ucsc.edu/goldenPath/help/bedgraph.html
SEG: https://cnvkit.readthedocs.io/en/stable/fileformats.html
Interact: https://genome.ucsc.edu/goldenPath/help/interact.html
bigInteract: https://genome.ucsc.edu/goldenPath/help/interact.html
MAF: https://docs.gdc.cancer.gov/Data/File_Formats/MAF_Format/
GWAS: https://software.broadinstitute.org/software/igv/GWAS
